# Dental Caries Diagnosis and Detection Using Neural Networks: A Systematic Review

**DOI:** 10.3390/jcm9113579

**Published:** 2020-11-06

**Authors:** María Prados-Privado, Javier García Villalón, Carlos Hugo Martínez-Martínez, Carlos Ivorra, Juan Carlos Prados-Frutos

**Affiliations:** 1 Asisa Dental, Research Department, C/José Abascal, 32, 28003 Madrid, Spain; javier.villalon@asisadental.com (J.G.V.); carlos.ivorra@asisadental.com (C.I.); 2 Department of Signal Theory and Communications, Higher Polytechnic School, Universidad de Alcala de Henares, Ctra, Madrid-Barcelona, Km. 33,600, 28805 Alcala de Henares, Spain; 3 IDIBO GROUP (Group of High-Performance Research, Development and Innovation in Dental Biomaterials of Rey Juan Carlos University), Avenida de Atenas s/n, 28922 Alcorcon, Spain; juancarlos.prados@urjc.es; 4 Faculty of Medicine, Universidad Complutense de Madrid, Plaza de Ramón y Cajal, s/n, 28040 Madrid, Spain; carlos.martinez@asisa.es; 5 Department of Medical Specialties and Public Health, Faculty of Health Sciences, Universidad Rey Juan Carlos, Avenida de Atenas, 28922 Alcorcon, Spain

**Keywords:** artificial intelligence, caries, images, detection

## Abstract

Dental caries is the most prevalent dental disease worldwide, and neural networks and artificial intelligence are increasingly being used in the field of dentistry. This systematic review aims to identify the state of the art of neural networks in caries detection and diagnosis. A search was conducted in PubMed, Institute of Electrical and Electronics Engineers (IEEE) Xplore, and ScienceDirect. Data extraction was performed independently by two reviewers. The quality of the selected studies was assessed using the Cochrane Handbook tool. Thirteen studies were included. Most of the included studies employed periapical, near-infrared light transillumination, and bitewing radiography. The image databases ranged from 87 to 3000 images, with a mean of 669 images. Seven of the included studies labeled the dental caries in each image by experienced dentists. Not all of the studies detailed how caries was defined, and not all detailed the type of carious lesion detected. Each study included in this review used a different neural network and different outcome metrics. All this variability complicates the conclusions that can be made about the reliability or not of a neural network to detect and diagnose caries. A comparison between neural network and dentist results is also necessary.

## 1. Introduction

Machine learning is an application of artificial intelligence (AI) that provides systems the ability to automatically learn and improve from experience without being explicitly programmed [1,2]. Machine learning needs input data, such as images or text, to obtain an output through a model.

Neural networks can be classified according to their typology or network structure or according to their learning algorithm. According to its topology, we can distinguish, as a characteristic of a network, the number of layers; the type of layers, which can be hidden or visible; input or output; and the directionality of the neuron connections. Depending on the typology, we can distinguish monolayer or multilayer networks.

According to its learning algorithm or how the network learns the patterns, we can distinguish as characteristics if it is supervised, unsupervised, competitive, or by reinforcement [3]. The model in supervised learning is trained, employing a labeled database. By contrast, the expected output is unknown in unsupervised learning [1,4]. Reinforcement learning is a model that falls between supervised and unsupervised learning.

The most common form of machine learning is supervised learning. To work with images, it is necessary, first, to collect a large data set of images, and second, to label each category in each image, in this case, with caries detected by a dentist. Then the training process begins. During the training process, the user/modeler feeds the data to network, it passes through the network, and an output is computed based on the current set of model weights. To obtain the best score of all categories, an objective function to measure the error is computed. Then the algorithm modifies its internal parameters to have the highest score of all categories. Finally, after training, the performance of the system is measured on a different set of images called a test dataset. The validation test serves to test the ability of the model to obtain good answers on new images (inputs) that it has never seen during the training process [5].

Convolutional neural network (CNN) is a type of deep and feedforward network. CNNs are designed to process data that come in the form of multiple arrays as images, and their architecture is composed of several stages.

Artificial intelligence is used in dentistry to identify and detect different variables from images, such as teeth, caries, and implants. Deep learning has been demonstrated to be a good collection of techniques to assist medical practitioners in medical fields such as radiology [6,7].

One of the most frequent activities in dental practice is to detect early caries lesions or to provide treatment preventing more invasive therapies [8]. The International Caries Detection and Assessment System (ICDAS) was developed by an international team of caries researchers to integrate several new criteria systems into one standard system for caries detection and assessment [9]. A workshop was organized to discuss and reach consensus about definitions of the most common terms in cariology [10]. Full agreement was obtained of the definition of dental caries:

“Dental caries is a biofilm-mediated, diet-modulated, multifactorial, non-communicable, dynamic disease resulting in net mineral loss of dental hard tissues. It is determined by biological, behavioral, psychosocial, and environmental factors. As a consequence of this process, a caries lesion develops”.

[10]

The definition of initial caries lesions was also obtained with full agreement and was defined as a frequently used term for noncavited caries lesions that refer to the stage of severity. Sound enamel/dentin was defined, with a 100% agreement, as a tooth structure without clinically detectable alterations of the natural translucency, color, or texture. However, other terms, such as secondary caries/recurrent caries, residual caries, or “hidden” caries, did not obtain full agreement [10]. During the first two sessions, ICDAS was formed and, afterwards, those criteria were revised, modified, and called ICDAS II [11].

The visual-tactile detection method is generally used in dental practice, followed by radiographic caries detection [8]. Bitewing radiography is the most frequent technique in carious lesion detection. Several studies have compared the performance of CBCT to conventional or digital intraoral radiography, histology, or micro CT for enamel and dentin caries detection [12,13]. The conclusion was that CBCT did not improve the accuracy of caries detection [14].

Dental caries is the most prevalent dental disease worldwide, and neural networks and artificial intelligence are increasingly being used in the field of dentistry. Many studies have contributed to the field of dental caries detection using neural networks with different dental images. This review aims to evaluate studies investigating caries detection with artificial intelligence and neural networks. This literature review analyzed in each study the type of image, the total image database and its characteristics, the neural network employed to detect caries, the exclusion criterion of images, and whether the database had been modified before the training process. Then, it was analyzed as to how caries were defined, what type of caries were detected, and the outcome metrics and values.

## 2. Materials and Methods

### 2.1. Review Questions

(1) What are the neural networks used to detect and diagnosis dental caries?
(2) How is the database used in the construction of these networks?
(3) How are caries lesions defined, and in which teeth are they detected?
(4) What are the outcome metrics and the values obtained by those neural networks?

### 2.2. Search Strategy

The research questions were elaborated considering each of the components of the PICO(S) [15] strategy research questions, which are explained as follows: (P) neural networks and caries detection; (I) caries definition and which teeth are detected; (C) studies with neural network are used to detect and diagnosis dental caries; (O) outcome metrics and values; (S) neural networks.

An electronic search was performed in the following databases up until 15 August 2020: MEDLINE/PubMed, Institute of Electrical and Electronics Engineers (IEEE) Xplore, and ScienceDirect.

The search strategy used is detailed in Table 1.

### 2.3. Study Selection and Items Collected

M.P.-P. and J.G.-V. performed the bibliographic search and selected the articles that fulfilled the inclusion criteria. Both authors collected all the data from the selected articles in duplicate and independently of each other. Disagreements between the two authors were reviewed using full text by a third author (J.C.P.-F.) to make the final decision. The references of the articles included in this study were manually reviewed.

The following items were collected: study (journal and year), type of image, total image database, database characteristics (pixels and examiners), neural network, image exclusion criteria, database modification (resized pixel), caries definition, caries type detected, teeth in which caries lesions were detected, outcome metrics (accuracy, sensitivity, specificity), and outcome metrics values.

### 2.4. Inclusion and Exclusion Criteria

The inclusion criteria were full manuscripts, including conference proceedings, that reported the use of neural networks for the detection and diagnosis of caries. There were no restrictions on the language or date of publication. Exclusion criteria were reviews, no dental caries application, no images, and no neural network employed.

### 2.5. Study Quality Assessment

The risk of bias from neural networks studies was evaluated by two of the authors (C.M.-M. and C.I.). To this end, the guidelines presented in the Cochrane Handbook [7] were followed, which incorporates seven domains: random sequence generation (selection bias); allocation concealment (selection bias); masking of participants and personnel (performance bias); masking of outcome assessment (detection bias); incomplete outcome data (attrition bias); selective reporting (reporting bias); and other biases.

The studies were classified into the following categories: low risk of bias—low risk of bias for all key domains; unclear risk of bias—unclear risk of bias for one or more key domains; high risk of bias—high risk of bias for one or more key domains.

### 2.6. Statistical Analysis

The mean, standard deviation (SD), median, and percentage were calculated for several variables. Statistical calculations were performed with IBM SPSS Statistics (SAS Institute Inc., Cary, NC, USA).

## 3. Results

### 3.1. Study Selection

**Figure 1:**
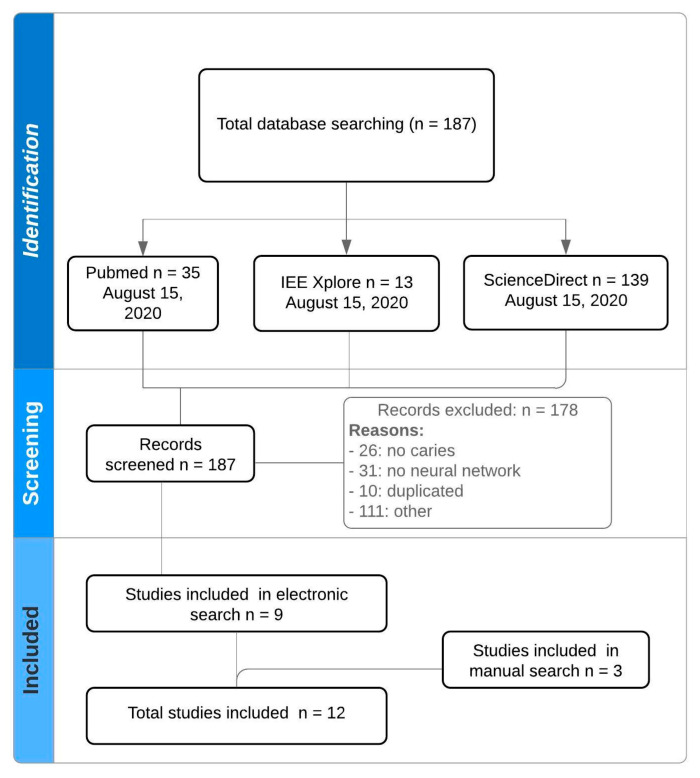
Flowchart.

Figure 1 details a flowchart of the study selection. All of the electronic search strategies resulted in 187 potential manuscripts. A total of 178 studies were excluded because they did not meet the inclusion criteria. Additionally, a manual search was carried out to analyze the references cited in ten of the articles that were included in this work. Finally, three more articles were incorporated from the manual search. In the end, a total of thirteen studies were analyzed.

### 3.2. Relevant Data about the Image Database and Neural Network of the Included Studies

Table 2 details the main characteristic of the studies included in the manuscript. Included studies were conducted between 2008 and 2020. All studies were published in English. Regarding the types of images, the most used were the periapical, the near-infrared light transilluminations, and the bitewings, each one appearing twice in each of the studies (16.66% in each study). The rest of the images used by a single study were panoramic radiographs, radiovisiography, intra-oral, in vivo with an intraoral camera and, and X-ray images (8.33%). Only two studies did not detail the type of image employed.

Image databases also varied from 87 to 3000 images, with a mean of 669.27 images, a standard deviation of 1153.76, and a median of 160 images. Seven (58.33%) of the included studies labeled the dental caries in each image by experienced dentists. Of those seven articles that used experts to indicate caries, five (71.42%) indicated that they use two experts (*n* = 2), one (14.28%) employed one expert, one employed four experts, and one employed 25 examiners. Three studies (25%) did not indicate the number of examiners.

Three studies detailed the exclusion criteria of the images. Six of the included studies detailed how images were standardized by resizing the number of pixels. 

### 3.3. Relevant Data about Caries of the Included Studies

Table 3 details the main characteristic of carious lesion detection and outcome metrics of the included studies. Three studies (23.07%) detailed how caries are defined in their studies. One study explained that a caries lesion was considered where a radiolucent area appears on the structure. The other two considered the caries definition that follows the ICDAS II classification system. Seven (53.84%) of the included studies detailed the type of caries detected: three studies detected occlusal caries, while the other four studies detected proximal, enamel, and dentinal lesions; pre-cavitated lesions; and initial caries. Six of the included studies did not detail the type of caries detected by their neural network or define what they considered as caries.

Regarding the teeth where caries lesions are detected, seven studies (53.84%) did not detail in which teeth caries were detected, four studies (30.7%) employed molar and premolar teeth, and two (15.38%) of the included studies used posterior extracted teeth.

Eight of the included studies analyzed accuracy, obtaining the following outcomes: a range from 68.57 to 99% (mean ± SD of 90 ± 7%, median of 89%), a precision range from 0.615 to 0.987 (mean ± SD of 0.801 ± 0.263), and an AUC from 0.74 to 0.971 (mean ± SD of 0.815 ± 0.1).

### 3.4. Study Quality Assessment

Evaluation of selection bias: All studies blinded image data.

Evaluation of performance bias: None of the studies indicated a blinding of staff or assessors.

Assessment of detection bias: All study results were blinded.

Evaluation of attrition bias: Not all of the studies reported complete results. Berdouses et al. [25] detailed all the results analyzed in the present review.

Evaluation of notification bias: Not all of the studies provided detailed information about the neural network parameters. Lee et al. [20] provided all the information.

**Figure 2:**
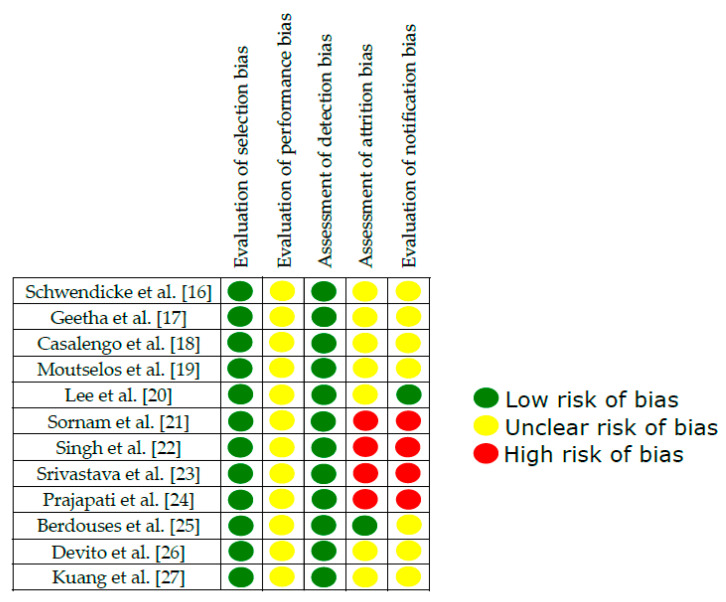
Assessment of risk of bias of included studies.

Figure 2 shows a detailed description of the risk assessment of bias in the included studies.

## 4. Discussion

The goal of this review is to visualize the state of the art of neural networks in detecting and diagnosing dental caries. The way in which each of the studies analyzes caries (definition, type, tooth), as well as the parameters of each neural network (type of network, characteristics of the database, and results), were studied.

A good definition of what is meant by caries and the type of caries lesions to be analyzed is essential to compare and analyze the results obtained in each study. Studies included in this review that detailed the use of ICDAS II obtained an accuracy between 80 and 88.9% (mean ± SD of 85.45 ± 6.29%), while the study that defined caries as a loss of mineralization of these structures (radiolucent) obtained an accuracy of 97.1%. However, 76% of the studies included in the present review did not detail how a caries lesion is defined.

Another bias factor is related to the training dataset. Images employed during the training process must be labeled by experts. Seven (58.33%) of the included studies indicated that examiners were used to label the images, although the experience and the number of those examiners varied from one study to another. Some studies analyzed the relation between caries detection and dentist experience. Bussaneli et al., concluded in their study that the experience of the examiner is not determinant to occlusal lesions in primary teeth but influenced the treatment decision of initial lesions [28]. However, when an artificial intelligence is trained with human observer’s scores, the system can never exceed the trainer and, therefore, the performance depends on the quality of the input.

An important fact for artificial intelligence technology is the overfitting. Burnham and Anderson describes “the essence of overfitting is to have unknowingly extracted some of the residual variation as if that variation represented underlying model structure” [29]. A model is overfitted when it is so specific to the original data that trying to apply it to data collected in the future would result in problematic or erroneous outcomes and therefore less-than-optimal decisions [30].

The included studies in this review that detailed the use of examiners to obtain an accuracy ranged from 80 to 97% (mean ± SD of 88.7 ± 8.55%). The best result was obtained in the study that used only one examiner, and, therefore, the same criteria in caries detection was always used, followed by the study where four experts analyzed the images. Finally, the worst result in terms of accuracy was obtained by the study with two examiners. Regarding the experience of the examiners, only one study detailed the number of years of experience. However, these results were not completely related to the number of examiners; other factors such as neural network, dataset, and caries definition must be kept in mind. In this sense, the results detailed in Table 3 must be analyzed with caution, since each of the networks used in the studies has a different purpose, which means that the results are not comparable between them. The data analyzed in a general way helps us to get an idea about what percentages of accuracy, on average, are obtained in caries detection and diagnosis studies using neural networks.

One of the limitations of this review is that studies using artificial intelligence with different tasks have been taken into account. This means that, although the studies obtain the same metrics, they cannot be compared with each other. The reason is that each artificial intelligence is designed for one thing that makes comparison of results impossible. From each to future reviews, it is recommended to include studies whose artificial intelligence has the same purpose. The use of a large dataset is crucial for the performance of the deep learning model. It is possible to improve the technical capability employing a technique called data augmentation. This technique artificially inflates the training database by oversampling or data warping. Oversampling creates synthetic instances and adds them to the training dataset. However, data warping transforms the existing images [31].

The study from Geetha et al. [17] presents the highest accuracy of all the studies included in the present review. However, this study is a special case because the authors built their own feature extractor, which is rare nowadays, and used a very shallow neural network with only one hidden layer. Authors of that study used only 105 images and did 10-fold cross-validation, and, therefore, their model was not evaluated on a hold-out test set.

A great variety of architectures has been found in the studies included in this literature review. ResNets are residual networks that are CNNs designed to allow thousands of convolutional layers. Mask R-CNN is an extension of Faster R-CNN by adding a branch for predicting segmentation masks on each Region of Interest (ROI) [5]. Semantic image segmentation is the task of classifying each pixel in an image from a predefined set of classes, which has several applications in medical images. Six (50%) of the included studies detailed that, before starting the training process, they homogenized the size of the images (Table 2).

Shokri et al., analyzed the effect of filters on detecting proximal and occlusal caries employing intraoral images and concluded that the lowest accuracy in caries diagnosis was noted for the detection of enamel lesions on original radiographs (52%). However, this in vitro study induced caries by a demineralizing solution, and therefore induced carious lesions were more regular than those that developed naturally [32]. Belém et al. [33] analyzed the accuracy of detection of subsurface demineralization by different imaging modalities and concluded that original images had an accuracy of 73% and a sensitivity of 62%. Kositbowornchai et al. compared the accuracy of detecting occlusal caries lesions on original images and obtained a mean Receiver Operating Characteristic (ROC) curve of 0.75 [34]. Here, two of the studies analyzed enamel lesions with an accuracy of 82% and a ROC curve of 0.717. The studies that analyzed occlusal lesions in this review obtained an accuracy of 80 and 88.9%, and a precision of 45.3%.

Several studies analyzed the precision in the detection of caries depending on the type of image used. Schwendicke et al., concluded in their systematic review that fluorescence-based images showed a significantly higher accuracy, sensitivity, and specificity in detecting initial lesions than conventional radiographic images, and generally that radiographic caries detection is especially suitable for detecting dentine lesions and cavitated proximal lesions [8]. Here, two of the studies employed near-infrared transillumination images and obtained similar outcome metrics to the other studies with different image types.

Supervised learning is one where the learning process of the algorithm from the training dataset can be considered to be a process supervised by a teacher. The correct answer is previously known, and the algorithm iteratively makes its predictions at the same time as it is corrected by the teacher. Seven (58.33%) of the included studies labeled the dental caries in each image by experienced dentists. However, in addition to knowing the number of examiners and their experience, it is very important to know what the intra-examiner agreement is, that is, to know if the examiner’s answers are the same if the categorization of the images is repeated a second time. It is also very important to know the inter-examiner agreement, that is, for the same image, how many examiners provide the same answer. None of the included studies mentioned the inter- and intra-examiner agreement. Intra- and inter-examiner agreement is evaluated by calculating Cohen’s Kappa. According to Bulman and Osborn [35], values of Cohen’s Kappa between 0.81 and 1.00 indicate almost perfect agreement.

Other graphical methods such as ROC (Receiver Operating Characteristic) curve or Bland-Altman plot can be employed to obtain information on those samples in which there is less agreement.

It is important to emphasize that manual labeling by experts provides a reference that is necessary for training and evaluating the model but does not necessarily represent ground truth [18]. The use of a histologic gold standard method is indispensable for the validation of a caries diagnostic method. None of the studies included in the present review mentioned the reference standard employed.

A quality analysis of the included studies was done using the Cochrane Handbook tool, which was employed to assess the risk of bias, concluding that in most domains, no data were given related to the transparency of the studies. This ensures that the data collected and analyzed have been managed in a controlled manner, avoiding all possible methodological errors. The criteria for allocation masking and randomization were not detailed in all of the studies, which is considered to be an unclear risk of bias. The present systematic review focused on the use of artificial intelligence in carious lesion diagnostic and detection; the bias is located in the lack of a reference standard and the inclusion of studies with different algorithms. However, the data presented above cannot be analyzed in isolation. Inter- and intra-examiner agreement must be taken into account in studies involving multiple examiners in order to obtain comparable and reliable results. This is a fundamental parameter to correctly define the variables that the neural network has to learn. That is, good agreement between examiners is essential to obtain good results once the image passes through the neural network. None of the studies using multiple examiners and included in this review detailed the concordance mentioned above in their respective studies. Neither was there a single parameter to compare the results obtained by the neural network, nor common parameters for the database. All these factors complicate the conclusions that can be made about the reliability or not of a neural network to detect and diagnose caries.

To be able to know if the neural networks give certain results, it is necessary to make a comparison with the results provided by the dentists, who should also have similar training and experience in order for comparisons to be made between them.

The diagnostic performance of artificial intelligence models varies between the different algorithms used and is still necessary to verify the generalizability and reliability of these models. For this, it would be necessary to use the ability to compare the results of the tasks of each algorithm before transferring and implement these models in clinical practice.

## Figures and Tables

**Table 1:** Search strategy.

Database	Search Strategy	Search Data
MEDLINE/PubMed	(deep learning OR artificial intelligence OR neural network *) AND caries NOT review	15 August 2020
IEEE Xplore	(deep learning OR artificial intelligence OR neural network) AND caries AND (detect OR detection OR diagnosis)	15 August 2020
ScienceDirect	(deep learning OR artificial intelligence OR neural network) AND caries AND (detect OR detection OR diagnosis)	15 August 2020

**Table 2:** Main characteristics of image database and neural network.

Authors	Neural Network Task	Image	Total Image Database	Database Characteristics (Pixels and Examiners)	Neural Network	Image Exclusion Criterion	Database Modification (Resized and Other)	Journal	Year
Schwendicke et al. [16]	Classification	Near-infrared light transillumination	226	Pixel: 435 × 407 × 3. Examiners: two (clinical experience, 8–11 years)	Resnet18, Resnext50	-	Resized pixel: 224 × 224	Journal of Dentistry	2020
Geetha et al. [17]	Classification	Intra-oral digital radiography	105	Pixel: Examiners: a dentist	ANN with 10-fold cross validation	-	Resized pixel: 256 × 256	Health Information Science and Systems	2020
Casalengo et al. [18]	Segmentation	Near-infrared transillumination	217	Pixel: Examiners: by experts	CNN trained on a semantic segmentation task	-	Resized pixel: 256 × 320	Journal of Dental Research	2019
Moutselos et al. [19]	Segmentation and classification	In vivo with an intraoral camera	87	-	DNN Mask R-CNN, which extends Faster R-CNN by adding an FCN for predicting object masks.	Teeth with hypoplastic and/or hypomineralized. Teeth with sealants on the occlusal surfaces.	-	Conf Proc IEEE Eng Med Biol Soc	2019
Lee et al. [20]	Classification	Periapical	3000	Pixel: Examiners: four calibrated board-certified dentists	CNN	Moderate-to-severe noise, haziness, distortion, and shadows. Full crown or large partial inlay restoration. Deciduous teeth.	Resized pixel: 299 × 299 Other: standardized contrast between gray/white matter and lesions.	Journal of Dentistry	2018
Sornam et al. [21]	Classification	Periapical	120	-	Feedforward Neural Network	-	-	IEEE International Conference on Power, Control, Signals, and Instrumentation Engineering (ICPCSI-2017)	2017
Singh et al. [22]	Detection	Panoramic radiographs	93	-	Radon Transformation (RT) and Discrete Cosine Transformation (DCT).	-	Resized pixel: 500 × 500	2017 8th International Conference on Computing, Communication and Networking Technologies (ICCCNT)	2017
Srivastava et al. [23]	Segmentation	Bitewing	3000	Pixel: Examiners: by certified dentists	FCNN (deep fully convolutional neural network)	-	-	NIPS 2017 workshop on Machine Learning for Health (NIPS 2017 ML4H)	2017
Prajapati et al. [24]	Classification	Radiovisiography	251	-	CNN	-	Resized pixel: 500 × 748	5th International Symposium on Computational and Business Intelligence	2017
Berdouses et al. [25]	Detection and classification	-	103	Pixel: Examiners: two	-	-	-	Computers in Biology and Medicine	2015
Devito et al. [26]	Detection	Bitewing	160	Pixel: Examiners: 25	Multilayer perceptron neural	-	-	Oral Med Oral Pathol Oral Radiol Endod	2008
Kuang et al. [27]	Segmentation	X-ray images	-	Pixel: 1000 × 800 Examiners: -	Back propagation Neural Network	-	-	Second International Symposium on Intelligent Information Technology Application	2008

CNN: Convolutional neural network.

**Table 3:** Main data about caries of the included studies.

Authors	Type of Study	Caries Definition	Caries Type Detected	Teeth	Outcome Metrics	Outcome Metrics Values
Schwendicke et al. [16]	in vitro	-	Occlusal and/or proximal caries	Premolar and molar	AUC, sensitivity, specificity, and positive/negative predictive values	0.74, 0.59, 0.76, 0.63, and 0.73
Geetha et al. [17]	in vitro	Loss of mineralization of these structures (radiolucent)	-	-	Accuracy, false positive rate, ROC, and precision	0.971, 0.028, 0.987
Casalengo et al. [18]	clinical	-	-	Upper and lower molars and premolars	IOU/AUC	72.7/83.6 and 85.6%
Moutselos et al. [19]		Classified from 1 to 6 using the ICDAS II classification system.	Caries on occlusal surfaces	-	Accuracy	0.889
Lee et al. [20]	in vitro	-	Dental caries, including enamel and dentinal carious lesions	Premolar, molar, and both premolar and molar	Accuracy, sensitivity, specificity, PPV, NPV, ROC curve, and AUC	82, 81, 83, 82.7, 81.4
Sornam et al. [21]	in vitro	-	-	-	Accuracy	99%
Singh et al. [22]	in vitro	-	-	-	Accuracy	86%
Srivastava et al. [23]	in vitro	-	-	-	Recall/Precision/F1-Score	0.805/0.615/0.7
Prajapati et al. [24]	in vitro	-	-	-	Accuracy	0.875
Berdouses et al. [25]	in vitro	ICDAS II	Pre-cavitated lesion and cavitated occlusal lesion	Posterior extracted human teeth	Accuracy	80%
Devito et al. [26]	in vitro	-	sound, enamel caries, enamel-dentine junction caries and, dentinal caries	Premolar and molar	ROC	0.717
Kuang et al. [27]	in vitro	-	Initial caries	-	Accuracy	68.57%

ICDAS: The International Caries Detection and Assessment System.
